# Molecular Mechanisms of Pathogenesis, Prevention, and Therapy of COVID-19: Summarizing the Results of 2022

**DOI:** 10.3390/ijms242216073

**Published:** 2023-11-08

**Authors:** Evgenii Gusev

**Affiliations:** Laboratory for Immunology of Inflammation, Institute of Immunology and Physiology of the Ural Branch of the Russian Academy of Sciences (IIP UB RAS), 620049 Ekaterinburg, Russia; gusev36@mail.ru

The aim of this Special Issue is to analyze the key patterns of the 2019 coronavirus disease pandemic (COVID-19), the biology of SARS-CoV-2 (severe-acute-respiratory-syndrome-related coronavirus 2, formerly 2019-nCoV), and the characteristics of the human body’s response to the invasion of this virus. The expected benefit is the ability to predict the most likely pathways of this infection and identify the most promising ways for preventing, diagnosing, and treating COVID-19 and its long-term consequences (long COVID). Despite the statistically confirmed positive results of mass specific prevention interventions using different types of vaccines [1], it has not been possible to completely stop the COVID-19 pandemic for several reasons. In particular, the subvariants of the SARS-CoV-2 omicron variant are now spreading rapidly, possibly due to altered antibody evasion properties resulting from their additional spike mutations [2]. This is also evidenced by the fact that the immune response to reinfection with omicron BA.1 tends to decline more rapidly than for the ancestral, alpha, and delta variants of SARS-CoV-2 [3]. The remarkable adaptive abilities of SARS-CoV-2 during parasitization in humans are especially noteworthy in this regard. This virus uses not only angiotensin-converting enzyme 2 (ACE2), the primary receptor for entry, but also several alternative receptors and co-receptors. Moreover, SARS-CoV-2 can induce abnormal variants of the immune response [4]. In this context, one of the contributions to this Special Issue demonstrates the potential involvement of the low-density lipoprotein receptor (LDLR) in the ACE2-independent internalization of SARS-CoV-2. Other studies show the protective role of antiviral CD56^+^ T cells (NKT-like cells) against COVID-19 but also the detrimental, depleting impact of CD8^+^ T cell subpopulations and natural killer (NK) cells on infection outcomes. One of the main causes of mortality among COVID-19 patients is the development of systemic inflammation and the associated cytokine storm phenomenon. As shown by Monserrat et al. in their work, a significant increase in blood cytokines, including IL-6, IL-8, IL-10, IL-15, IL-18, IL-27, and M-CSF, in addition to soluble cytokine receptors such as sIL-1RI, sIL1RII, sTNF-RII, and sIL-2Ra, precipitates the fatal outcome of this disease [5]. Other articles in this Special Issue demonstrate that high levels of TNF-α and IFN-γ in the development of a CoV-associated “cytokine storm” have negative impacts on patient survival. Neutrophil extracellular traps (NETs) and oxidative stress at the center of infectious inflammation are thought to be useful for innate immune defense against pathogens. However, as shown by Hosseini et al., forced uncontrolled NET formation and lipid peroxidation in the lungs during COVID-19 infection can result in a critical complication of viral pneumonia, namely, the development of acute respiratory distress syndrome [6]. Another paper in this Special Issue shows that the failure of antioxidant protection in severe COVID-19 cases may be associated with a polymorphism of the NFE2L2-KEAP1 genes, which are involved in cellular protection against oxidative stress. In preclinical and clinical studies, mesenchymal stem cell (MSC) therapy has been shown to be safe and, in some cases, effective in terms of alleviating the severe complications of COVID-19 [7]. Further support for this promising direction in COVID-19 therapy is provided by original papers contributed to this Special Issue. As demonstrated by these and other original and review papers in this Special Issue, effective treatment of COVID-19 requires a multi-stage, stage-specific approach to achieve a complete cure.

A noteworthy aspect of COVID-19 is the phenomenon of long COVID, which presents a significant challenge for the public health system in most countries around the world [8,9]. Long COVID symptoms can last for up to 12 months or even longer. At the same time, they remain largely unexplained and under-researched, and it is unclear how long the symptoms of long COVID can persist. To address this emerging public health crisis, new strategies are urgently needed. There is evidence suggesting that long COVID is not a specific syndrome or some other clinical definition; rather, it is a group of pathologies associated with both canonical and non-canonical types of inflammation. These variants of non-canonical inflammation include not only acute systemic hyperinflammation, which is a life-threatening condition, but also low-grade inflammation [10]. At present, there are not many data indicating the pathogenetic relationship between long COVID and the onset or progression of local and systemic variants of low-grade inflammation [11,12]. Chronic low-grade inflammation, in turn, leads to proinflammatory activation and the dysfunction of microglia and other brain cells with excessive cytokine release, causing various neuroinflammatory complications [13]. At the same time, many neuropsychological and cognitive disorders are also attributed to long COVID [14]. Of particular concern among these complications is the pathogenetic association of long COVID with myalgic encephalomyelitis/chronic fatigue syndrome (ME/CFS) [15,16] as well as its relationship with the onset and progression of neurodegenerative disorders [17]. One of the original articles in this Special Issue confirms the association of COVID-19 with Parkinson’s disease progression. Endothelial dysfunction associated with low-grade inflammation plays a central role in the development of hypertension and atherosclerosis and their complications, including vascular thrombosis and stenosis [18]. Persistent microvascular endotheliopathy associated with long COVID can potentially contribute to these and other serious complications [19]. Clearly, the importance of studying long COVID will continue to grow for a long time to come. Although the head of the World Health Organization (WHO) has declared an end to the COVID-19 global health emergency, this disease remains a global threat. To manage such pandemics in the future, it is imperative to develop the proper strategies and abilities to protect human life [20].

P.S.: This editorial briefly reflects the content of all 20 papers in this Special Issue. Only two publications in this Special Issue are presented in the references [5,6]. For more details, the data from the other publications of this Special Issue can be found at https://www.mdpi.com/journal/ijms/special_issues/COVID19_2022, accessed on 3 July 2022.
